# Are Junior Tennis Players Less Exposed to Shocks and Vibrations than Adults? A Pilot Study

**DOI:** 10.3390/s24247999

**Published:** 2024-12-14

**Authors:** Tom Le Solliec, Christophe Hautier, Robin Gassier, Robin Trama, Benoit Gilbert, Lin Song, Qingshan Zhang

**Affiliations:** 1 Université de Lyon, UCBL1 Laboratoire Inter Universitaire de Biologie de la Motricité, EA 7424, 69100 Villeurbanne Cedex, France; tom.ls@hotmail.fr (T.L.S.); christophe.hautier@univ-lyon1.fr (C.H.); r-gassier@live.fr (R.G.); 2 Babolat VS, 69009 Lyon, France; bgilbert@babolat.com; 3 Human Performance Laboratory, Faculty of Kinesiology, University of Calgary, Calgary, AB T2N 1N4, Canada; trama.robin@gmail.com; 4 School of Athletic Performance, Shanghai University of Sport, Shanghai 200438, China; songlinccc@hotmail.com

**Keywords:** overhead sport, muscular activation, shock, vibration, junior

## Abstract

This study investigated muscle activation, shocks, and vibrations of the upper extremities during tennis serves between junior and adult tennis players. Thirty-five well-trained tennis players (15 juniors and 20 adults) performed 10 maximal successful tennis serves. Two triaxial accelerometers recorded the shock and vibration on the racket and the hand on the dominant side. Eight surface EMG electrodes were also used to measure the arm muscles’ activities. Linear mixed models were used to test the fixed effect of age on muscular activation and vibration. Statistical non-Parametric Mapping was employed to make statistical inferences on the EMG and accelerometer data obtained from the continuous wavelet transform. Comparing EMG parameters between junior and adult players reveals similar upper limb intermuscular coordination. The junior players experienced lower racket and hand vibration amplitudes, which were partially explained by a lower ball velocity. This study revealed that young players showed no difference in EMG parameters in the tennis serve but were as exposed to shocks and vibrations as adults when compared based on a given speed and a given handgrip force. These vibrations apply to an immature skeleton, which can increase the risk of injuries caused by overuse. In addition, differences in the racket vibration frequency provide original knowledge to engineers who need to develop innovative sports equipment for tennis.

## 1. Introduction

Playing tennis has beneficial effects on health that have been clearly demonstrated [1]. In junior and adult players, tennis improves neuromuscular function, cardiovascular health [2], and bone mineral density [3]. In contrast, it has been shown that tennis can also cause overuse pathologies due to the mechanical constraints generated by this sport, mainly affecting the muscles, joints, and tendons [4,5]. Numerous pieces of evidence revealed that the magnitude of shock and its repetitive transmission to the musculoskeletal system (e.g., muscle, bone, and musculotendinous structures) could induce muscular fatigue to enhance the potential risk of musculoskeletal overuse injuries [6,7,8,9]. Considering that racket shock stimulates vibration, some recent studies investigated the effects of tennis racket shock on vibration transmission to the human forearm. For instance, ref. [10] observed that the amount of vibration at the racket and wrist was significantly amplified when playing velocity increased during forehand drives in tennis players [10].

Although the epidemiology of tennis shows a low incidence of chronic pathologies in junior players compared to adults, they may suffer from different overuse pathologies due to microtraumas like rotator cuff tendinitis, epicondylitis, chronic muscle strain, growth plate injuries, and stress fractures [4,11]. Previous evidence showed that the number of injuries players suffer increases with age [12]. For instance, the shoulder joint is often affected in young players [4], as demonstrated by the 24% of junior players with previous shoulder injuries [13]. It may also be considered that the repetition of muscular contractions, shocks, and vibrations could induce a latent inflammatory state that is not detectable in junior players but able to develop over time until favoring the appearance of neuromuscular and tendon overuse pathologies [4,14]. It seems likely that the increase in injuries after puberty may result from the accumulation of muscular constraints and shocks experienced by junior players during their training as athletes.

Furthermore, considering that the muscle contraction level tends to dampen the vibration as soon as an impact is initiated, it is worth observing the muscle activity during the impact. One primary mechanism to reduce soft tissue vibration is ensuring muscle activation is higher during the impact phase, allowing the muscle to increase muscle stiffness. During the acceleration of a tennis serve, the subscapularis and Pectoralis Major muscles are activated at over 110% of the maximal muscular activation during the acceleration phase of the serve [15], and muscle activation occurs around the scapulohumeral articulation in the serve, indicating that there is a difference in the activation of muscles around the scapulohumeral articulation in the typically accomplished tennis serve following timing sequence (i.e., scapular stabilizers before rotator cuff) [16]. Despite the studies that have reported muscular activity in adult tennis players during different fundamental actions [17], a few studies have only investigated muscular activities of the upper limb during junior tennis serves [18]. It could be suspected that the racket impact might transfer different levels of vibration to the arm muscle, and the vibration damping could be distinct due to the neuromuscular capacity being different between junior and adult players [19]. In particular, researchers have yet to investigate both arm and shoulder muscle activity and junior muscle activation during the serve action. As a result, it is essential to quantify the muscle activity of the forearm in junior tennis players compared to adult players, which permits the comparison of the potential risk of injury between adult and junior tennis players to be qualified. Also, the initial input of the shock (e.g., peak racket acceleration) and the capacity to damp the impact (e.g., setting time) to reduce the soft tissue vibration allow us to better understand the risk of injury between juniors and adults.

Therefore, the purpose of the present study was to compare the level of muscle recruitment, shocks, and vibrations during the tennis serve in juniors and adults. It was hypothesized that (*i*) junior tennis players suffer lower shock and vibration; (*ii*) the activation of the upper limb muscles is higher in adult tennis players than junior players; and (*iii*) therefore, adult tennis players damp the vibration less than junior tennis players.

## 2. Materials and Methods

### 2.1. Participants

A priori sample size estimation was calculated using G*Power 3.1.9.7 (Hein-rich-Heine-Universität Düsseldorf, Düsseldorf, Germany) based on an ANOVA method. The required sample size was calculated as 30 participants, based on an expected effect size of η^2^ = 0.25 [17], a statistical power of 0.85, and a significance level (α) of 0.05 (ref). A total of thirty-five tennis players (15 male juniors and 20 adults, 11.6 ± 1.0 vs. 19.0 ± 0.7 years; 151.1 ± 7.8 vs. 177.7 ± 8.1 cm, 41.3 ± 10.1 vs. 69.5 ± 8.1 kg; tennis training per week: 3.8 ± 1.0 vs. 3.5 ± 1.3 h; International Tennis Number (ITN): 7–10 vs. 4–5) volunteered to participate in this study. The players had been free from injury for six months before the experiment. Parents and participants signed informed consent documentation. This study was conducted according to the World Medical Association Declaration of Helsinki. This study was approved by the local ethics committee of Lyon 1 Claude Bernard University.

### 2.2. Experimental Procedures

**Figure 1:**
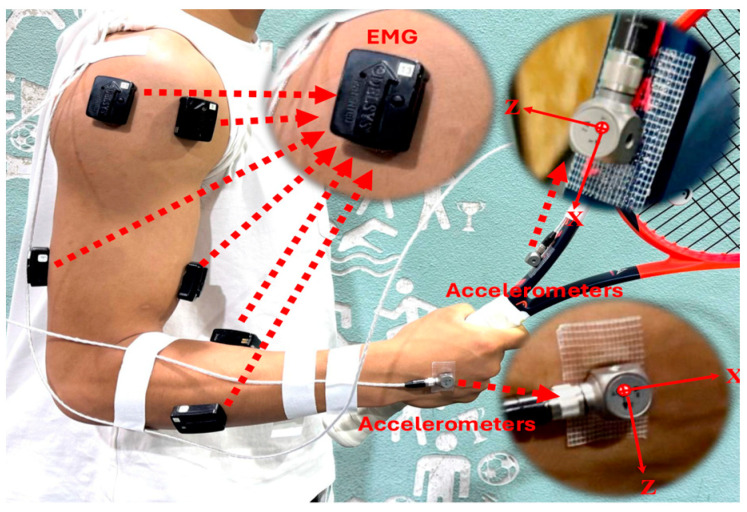
Indicates the location of two triaxial accelerometers and eight surface EMG electrodes.

**Figure 2:**
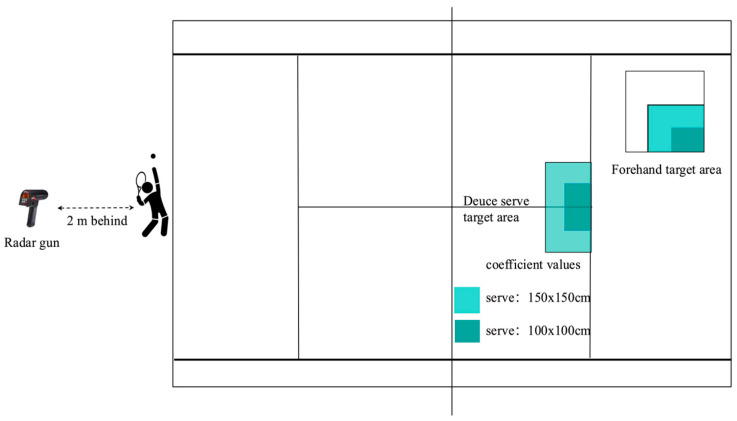
Representation of the tennis serve test setup. The dark and light grey zone represents the target dimension for junior and adult players, respectively.

A cross-sectional design investigated the difference between adult and junior tennis players in shock and soft tissue vibration. All the participants performed a 12-min standardized warm-up (i.e., 3 min of running, 5 min of forehand drive rallies against an opponent, and 4 min of serving). Afterward, a hand grip test was performed to obtain the maximal isometric of the Flexor Carpi and grip. And then, eight surface electromyographic (sEMG) electrodes (Delsys TrignoTM Wireless EMG System, bipolar Ag: AgCI surface, 2 cm inter-electrode distance) (Delsys, Inc., Boston, MA, USA, sample rate: 2000 Hz) were fixed on the Pectoralis Major (PM), Upper Trapezius (UT), Deltoideus Anterior (DA), Deltoideus Medius (DM), Biceps Brachii (BB), Triceps Brachii (TB), Flexor Carpi (FC), and Extensor Carpi (EC) muscles of the dominant arm, following the SENIAM guidelines [20] (Figure 1). Additionally, two triaxial accelerometers (Dytran Instrument Inc., Chatsworth, CA, USA, ±1000 m·s^−2^, frequency: 2000 Hz) were taped to the back of the hand and to the throat of the racket in the dominant arm before the testing. All signals were synchronized with LabVIEW 3.0 (National Instrument, Austin, TX, USA) (Figure 1). After securing the equipment to the participants, each participant performed Maximum Voluntary Isometric Contraction (MVIC) for the Pectoralis Major, Upper Trapezius, Deltoideus Anterior, Deltoideus Medius, Biceps Brachii, Triceps Brachii, Flexor Carpi, and Extensor Carpi muscles according to the previous study [17]. The participants performed three isometric warm-up trials (approximately 50% of maximal), followed by three 4 s MVIC trials separated by 60 s. After 5~6 min of recovery, each participant performed at least fifteen tennis serves, considered as familiarization. Based on the differences in International Tennis Number levels, we used a target area of 150 × 150 cm for juniors and 100 × 100 cm for adults, both positioned over the “T.” [17] (Figure 2). A total of 10 successful tennis serves for each participant were used for data analysis. All participants in the same group used the same racket for the test session, i.e., a Babolat Pure Drive Junior 25 (weight: 240 g unstrung, length: 635 mm, balance: 315 mm, head size: 630 cm^2^, rigidity: 60 RA, and composition: graphite) for juniors and a Babolat Pure Drive (weight: 300 g unstrung, length: 685 mm, balance: 320 mm, head size: 645 cm^2^, rigidity: 72 RA, and composition: graphite) for adults.

### 2.3. Measurement and Data Processing

#### 2.3.1. Hand Grip Force

The hand grip force was measured by a Camry electronic hand-held dynamometer (Model EH101, Zhongs-han Camry Electronic Co., Ltd., Shenzhen, China).

#### 2.3.2. Ball Velocity

Peak ball velocity was measured in real time using a radar gun (Stalker Pro II, Stalker Radar, Plano, TX, USA), which was positioned approximately 2 m behind the player and at a height equal to the racket head at the point of the ball contact. The mean ball velocity of ten successful serves (i.e., higher ball velocity) was used for data analysis.

#### 2.3.3. Shock and Soft Tissue Vibration

The time–frequency domain of the shock (i.e., racket accelerometer) and vibration (i.e., hand accelerometer) signals was investigated with a continuous wavelet transform (CWT), using a filter bank of 273 Morse wavelets, with a symmetry parameter equal to 3 and a time–bandwidth product equal to 60 [21]. The time resolution of the 273 scaled wavelets extended from 0.6 (10 Hz) to 0.013 s (500 Hz). The peak frequency of the wavelet was used to determine the pseudo-frequency of each wavelet. The 3 dimensions of the CWT results, including the frequency, time window, and amplitude, were presented as maps, which were linearly interpolated due to the logarithmical spacing of the frequencies to allow a regular frequency response. The modulus of the coefficient given by the wavelet transform was computed for each axis of each accelerometer, and the power of the signal for each time and frequency was determined by calculating the norm of the three axes. The amplitude spectrum ranging from 10 to 500 Hz was analyzed. The median frequency was calculated as the frequency that split the coefficient map into two equal parts. The damping was calculated using the setting time (ST) as the period between the peak amplitude and when the amplitude reached 10% of this maximum [22]. The total amplitude was computed as the map’s double integral by the time and frequency.

#### 2.3.4. EMG Analysis

A 4th-order Butterworth band-pass filter between 20 and 500 Hz was applied for the raw EMG data. A 500 ms time window was used for analyzing eight targeted muscles, using 300 ms before and 200 ms after the ball/racket impact corresponding to the acceleration (drive) and braking phases (backswing) [23]. After that, the envelope of the filtered signal was calculated by the root mean square (RMS) value with a 100 ms window length. Afterward, the signs were normalized by the MVICs, expressed as a percentage (%) of maximal activation. A mean value was calculated for each subject using the ten tennis serves kept for the analysis. The maximal EMG (EMG_max_) and average EMG (EMG_mean_) corresponded to the maximal and mean muscular activation during the serve, respectively. Integrated EMG (iEMG) is calculated as the area under the curve of the RMS EMG signal, that is, the mathematical integral of the absolute value of the RMS EMG signal. A continuous wavelet transform (CWT) was made on filtered EMG signals to obtain the power spectrum ranging from 20 to 500 Hz to investigate the time–frequency domain, using a Morse mother wavelet with γ = 3 and *P*^2^ = 60 [21,24]. The median frequency (MF) was defined as the frequency that split the power spectrum in half.

#### 2.3.5. Statistics Analysis

Prior to the statistical analysis, the normality, homoscedasticity, and linearity of the model residuals were graphically controlled. Linear mixed models were used to test the effect of age (fixed effect: adult vs. junior) and racket on variables of muscular activation, hand grip force, racket, and hand vibration [25] Likelihood ratio tests were conducted by testing the full model against the model without the effect tested to obtain the *p*-values. The original alpha error (5%) was corrected with a sequential Bonferroni correction for each group test to prevent false-positive results, and the direction of the differences was determined from the mean of each group. All the statistical calculations were performed using R software (R 3.5.0, RCore Team, Vienna, Austria). The significance level was set at 0.05.

In addition, Statistical non-Parametric Mapping (SnPM) analysis was employed to make statistical inferences on the EMG and accelerometer data obtained from the continuous wavelet transform using the MATLAB 2023 package fctSnPM [26,27]. Concerning the EMG, the mean temporal representation of normalized EMG was considered for the analysis. The 300 ms before the impact up to 200 ms after the impact was investigated, and this duration corresponded to 1000 nodes. Student’s *t*-Test (adult vs. junior) with an alpha error at 0.05/8 was used to analyze temporal continuums as one test was performed for each electrode (eight). Concerning the accelerometers, a similar methodology as that of [27] was employed, except that the maps were linearly interpolated by 0.02 s in the time domain and by 3 Hz in the frequency domain. The 200 ms after the impact was analyzed and this duration was interpolated to 100 samples. Thus, the number of nodes of the maps was 100 [time] × 164 [frequency] = 16,400 nodes. Student’s *t*-Test (adult versus junior), with an alpha error of 0.05/2, was used to analyze map continuums because one test was performed for each accelerometer. Ten thousand permutations were calculated to define the threshold of the statistical tests. Therewith, effect sizes (ESs) were calculated with Hedges’ g for each node of the power maps and each variable. 

## 3. Results

### 3.1. Hand Grip Force

Adult players presented a higher hand grip force than junior players (40.9 ± 8.3 vs. 23.5 ± 5.8 kg, η^2^ = 0.269, *p* < 0.01, Table 1).

### 3.2. Peak Ball Velocity

Junior players presented a 31% lower peak ball velocity compared to adult players (87.0 ± 15.0 vs. 127.6 ± 28.5 km·h^−1^, η^2^ = 0.247, *p* < 0.01, Table 1).

### 3.3. Shock and Soft Tissue Vibration

**Figure 3:**
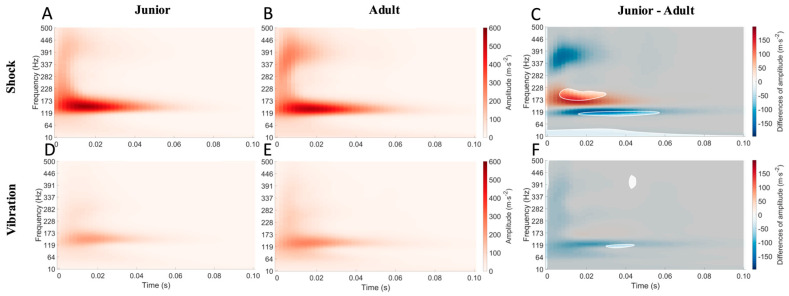
Mean maps of the time−frequency power of vibration on junior (**A**) and adult (**B**) rackets and junior (**D**) and adult (**E**) hands and the SPM differences of amplitude between junior and adult players on the racket (**C**) and the hand (**F**). Blue areas mean a higher amount of energy for adults compared to junior players and vice versa for red areas.

Considering the racket vibrations, the junior players presented a lower median frequency (179.65 ± 10.67 vs. 200.57 ± 45.46 Hz, η^2^ = 0.051, Table 1) and 21% lower peak acceleration (2246.71 ± 505.07 vs. 2849.02 ± 499.75 m.s^−2^, η^2^ = 0.11, Table 1) compared to adult players. In contrast, no significant differences were observed on the other racket vibration variables (all *p* > 0.05, Table 1). Furthermore, the junior players showed 33% lower peak vibrations of the hand compared to adult players (1068.68 ± 427.55 vs. 1609.5 ± 556.85 m.s^−2^, η^2^ = 0.091, Table 2). In contrast, no other significant difference was found in other hand vibration parameters (all *p* > 0.05). Moreover, the SPnM results indicated that the adult players showed significantly more energy for the shock at the zone of medium (between ~170 and ~220 Hz) and low-frequency (between ~90 Hz and ~110 Hz) domains from 0.01 s to 0.06 s (see Figure 3). In contrast, there were only small significant differences in the zone of high- (between ~390 and ~450) and low-frequency domains (between ~110 Hz and ~120 Hz) in the vibration from 0.03 s to 0.045 s between the adult and junior athletes (see Figure 3).

### 3.4. Muscular Activation of the Upper Limb

**Figure 4:**
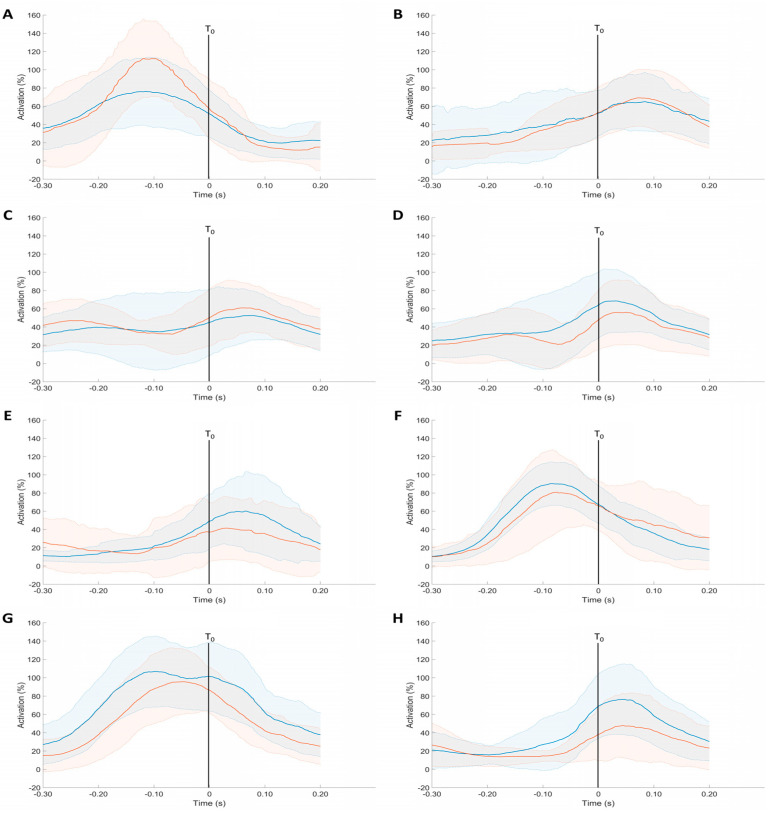
Mean (±standard deviation) muscular activation of junior (blue) and adult players (red) of Pectoralis Major (**A**), Upper Trapezius (**B**), Deltoideus Anterior (**C**), Deltoideus Medius (**D**), Biceps Brachii (**E**), Triceps Brachii (**F**), Flxor Carpi (**G**), and Extensor Carpi (**H**) during the serve (T0, corresponding to the ball impact).

Adult players presented a higher median frequency of Deltoideus Medius (152.72 ± 27.12 vs. 130.49 ± 24.86, η^2^ = 0.058, *p* < 0.05) (Table 2). However, junior and adult players showed no differences in maximal and meant EMG levels before and after the ball’s impact on the racket (all *p* > 0.05) (Table 3). Moreover, the SPM analysis did not reveal significant differences in the EMG parameters between the junior and adult players (Figure 4). The activation of the FC, EC, BB, TB, DM, DA, PM, and UT muscles started in a fixed order in adults and juniors. There was maximal activation of PM, FC, and TB at 0.10 s before hitting the ball and of UT, EC, BB, DM, and DA at 0.05 s after the impact. The PM, FC, and TB activation levels dropped quickly after the impact, while the other muscles remained activated. Interestingly, Flexor Carpi muscles were extensively and almost continuously activated throughout the movement in the two groups (Figure 4).

## 4. Discussion

To the best of our knowledge, this is the first study to compare racket shock and hand vibration parameters and muscle recruitment during the serve between junior and adult tennis players. The results tended to indicate that children are as exposed to vibrations as adults if we compare a given ball speed and a given handgrip force. Additionally, the muscular activity and recruitment did not indicate a significant difference between junior and adult players. Specifically, the method used to compare racket shocks and hand vibrations between adults and juniors has never been used previously in the literature. This very robust method enables the analysis of transitory vibrations in the time–frequency domain. Moreover, the SnPM statistical method provides a unique opportunity to compare vibration signals between adult and junior players.

### 4.1. Shocks and Vibrations Between Junior and Adult Players

The vibration amplitude recorded in the present study for adult players (2849.0 ± 722.2 m·s^−2^) was similar to previous results [28,29,30], which ranged from 2500 to 3200 m·s^−2^. Concerning the comparison of shocks and vibrations induced by the serve, the present results indicated that the junior players’ rackets vibrated less than the adult players’ rackets. These results may be due to the interaction between the ball velocity (87.0 vs. 127.6 km·h^−1^) and grip force (23.5 vs. 40.9 kg) between the junior and adult players. It is known that the vibration characteristics are dependent on shock exposure and muscle strength. The present finding indicated that junior players displayed approximately 21% lower total amplitude racket vibration (Table 2, Figure 3) and 33% lower hand vibrations, which is in line with the 31% lower peak ball velocity recorded in serves. It seems that shocks and vibrations depend largely on ball velocity. However, it should be kept in mind that the hand grip force was 42% lower in juniors and that these vibrations apply to an immature skeleton more exposed to trauma such as stress fractures and joint and muscle damage. It could also be supposed that the higher grip strength could reduce vibration exposure from the racket and benefit from the hand’s mechanical stability following the higher grip force. Thus, the junior players appeared to need to enhance their grip strength, which allowed them to be more efficient in damping racket vibrations.

Concerning the frequency, it is more difficult to strictly compare our results to previous ones since we calculated the median frequency. In contrast, other studies reported the mean frequency [31] and the fundamental frequency [10]. However, the frequency modes of vibration calculated from the wavelet transform are similar to the published results [30,32]. Additionally, the vibration duration obtained in this study is similar to the damping time reported by Rogowski et al. [10] (~0.46 ms) and Stroeder et al. [33] (~0.52 ms). Thus, the measurement and calculation methods used in the present study provided reliable results and enabled the comparison of juniors and adults. Interestingly, damping time was similar in the two groups, suggesting that juniors could implement the muscle-tuning paradigm. They may adjust the mechanical muscle properties [34,35] to modify the natural frequency of musculotendinous structures and increase the damping properties [36]. Further studies are needed to explore this hypothesis to help design rackets for juniors adapted to their physical capabilities and vibration tolerance.

### 4.2. EMG Between Junior and Adult Players

The results of the present study indicated that regardless of the muscle, the average EMG did not show a significant difference between junior and adult tennis serves (Figure 4). This original result suggests that in a well-trained junior (3.80 h/week), the complex intermuscle coordination is designed and established for the adult. The analysis of the EMG could be conducted considering the order of muscle activation, named activation sequences, and the level of muscle activation expressed as a percentage of maximal EMG intensity for each muscle and each subject [17]. The SnPM method provided information on these two parameters in a unique analysis and demonstrated that no difference could be evidenced between the two groups. This is the first time that the EMG was analyzed in junior players, so no comparison could be made with the literature. However, the EMG sequence obtained in adult tennis serves in this study is similar to that published by Ryu et al. [15]. Such an EMG aligns with the pattern reported by Kibler et al. [16]. Considering the level of muscle activation, i.e., the maximal relative EMG value, it appears that PM, FC, UT, and TB were more highly activated than the other muscles. The muscles strongly activated during the tennis serve are likely to be stressed and develop more than the others. This may lead to pathologies linked to an imbalance between agonist and antagonist muscles around a joint [37]. For example, repeated strong concentric and eccentric contractions of shoulder muscles [15] can result in anteroposterior force imbalance [38] and glenohumeral internal rotation deficit, which may, in turn, cause pathologies in the shoulder joint [4]. The fact that juniors present, from the age of 12, a strong activation of the PM during the serve gesture can potentially be linked to pathologies of the shoulder that can appear at an early age and generally progress with age and years of practice [38,39]. These results confirm that the shoulder is a highly solicited joint in tennis [4], especially during tennis serve, even in young players. The predominant role of the shoulder joint should be kept in mind to develop further studies that may help to understand and prevent the progressive development of injuries during the careers of talented young players. Thus, this can be explained by the intensity of play, which increases with age and level.

## 5. Limitation

This study presents limitations that warrant discussion. The first limitation of this study lies in the relatively small number of subjects; this may limit the transfer of these results to a larger population. Also, the present study did not examine neuromuscular fatigue, which could impact muscular activities and shock properties [40]. However, it should be kept in mind that these subjects were well trained and presented a good performance in relation to their age. The second limitation lies in the number of tennis serves that were considered. However, it was difficult for junior players to perform more than 15 good first serves.

## 6. Conclusions

The present study showed that young tennis players had a similar muscular activation to adults. When compared for a given speed and a given handgrip force, we can conclude that children are as exposed to shocks and vibrations as adults. However, these vibrations apply to an immature skeleton, which can increase the risk of overuse injuries. This study is the first comparison of muscle recruitment, shocks, and vibrations during the serve task between junior and adult tennis players. These findings provide original knowledge for sports medicine to trace the source of the incidence of chronic pathologies and for engineers to develop innovative sports equipment for tennis players. Adapting the game format and/or equipment seems an important way for young players to avoid exposure to shock and vibration stresses too early. Further studies are needed to assess these parameters in young players of different ages and stages of growth to understand the evolution of risk exposure during a young player’s career and secondly to determine if these vibrations are dangerous for the child’s anatomy or if this may reduce player performance by increasing muscle fatigue.

## Figures and Tables

**Table 1:** Mean ± SD of grip force, ball velocity, peak acceleration, median frequency, and setting time of racket impact and hand vibration. **: *p* < 0.01.

		Adult	Junior			
	Variable	Mean ± SD	Mean ± SD	Difference [95%]	Effect Size (η^2^)	Qualitative Inference
	Grip force (N)	40.91 ± 8.30	23.17 ± 6.41	−17.74 [−24.10; −11.38]	0.269	Large
	Ball velocity (m/s)	120.48 ± 33.11	89.19 ± 27.76	−31.28 [−57.27; −5.30]	0.247	Medium
Shock	PA (m^2^.s^−1^)	2849.02 ± 499.75	2246.71 ± 505.07	−602.31 [−952.98; −251.65] **	0.11	Medium
	MF (Hz)	200.57 ± 45.46	179.65 ± 10.67	−20.93 [−42.79; 0.93]	0.051	Small
	ST (s)	0.05 ± 0.01	0.04 ± 0.01	0 [−0.01; 0]	0.013	Small
Vibration	PA (m^2^.s^−1^)	1609.5 ± 556.85	1068.68 ± 427.55	−540.83 [−879.39; −202.26] **	0.091	Medium
	MF (Hz)	153.34 ± 15.09	152.58 ± 10.3	−0.76 [−9.51; 7.98]	0	Small
	ST (s)	0.04 ± 0.01	0.04 ± 0.01	0 [0; 0]	0.001	Small

**Table 2:** Mean ± SD of median frequency (MF), mean EMG (EMG_mean_), integral EMG (iEMG), and maximal EMG (EMG_max_) of Pectoralis Major (PM), Upper Trapezius (UT), Deltoideus Anterior (DA), Deltoideus Medius (DM), Biceps Brachii (BB), Triceps Brachii (TB), Flexor Carpi (FC), and Extensor Carpi (EC) during acceleration phases (Acc). *: *p* < 0.05.

			Adult	Junior			
Muscle	Phase	Variable	Mean ± SD	Mean ± SD	Difference [95% CI]	Effect Size (η^2^)	Qualitative Inference
BIC	Acc	MF (Hz)	124.16 ± 21.24	126.93 ± 21.14	2.775 [−12.69; 18.24]	0.001	Small
BIC	Acc	EMG_mean_ (%)	20.25 ± 25.47	21 ± 8.6	0.748 [−12.59; 14.09]	0	Small
BIC	Acc	iEMG (AU)	6.07 ± 7.64	6.3 ± 2.58	0.226 [−3.78; 4.23]	0	Small
BIC	Acc	EMG_max_ (%)	47.6 ± 36.94	52.21 ± 25.72	4.612 [−18.05; 27.27]	0.001	Small
DANT	Acc	MF (Hz)	137.7 ± 26.38	126.31 ± 18.96	−11.386 [−27.23; 4.45]	0.02	Small
DANT	Acc	EMG_mean_ (%)	26.89 ± 17.75	37.82 ± 24.69	10.935 [−5.03; 26.9]	0.02	Small
DANT	Acc	iEMG (AU)	8.06 ± 5.33	11.34 ± 7.41	3.278 [−1.51; 8.07]	0.028	Small
DANT	Acc	EMG_max_ (%)	63.07 ± 36.36	82.24 ± 35.11	19.174 [−6.23; 44.58]	0.024	Small
DMED	Acc	MF (Hz)	152.72 ± 27.12	130.49 ± 24.86	−22.226 [−40.71; −3.74] *	0.058	Small
DMED	Acc	EMG_mean_ (%)	40.4 ± 15.18	35.74 ± 28.33	−4.656 [−21.61; 12.3]	0.004	Small
DMED	Acc	iEMG (AU)	12.12 ± 4.55	10.72 ± 8.5	−1.394 [−6.48; 3.69]	0.005	Small
DMED	Acc	EMG_max_ (%)	72.31 ± 26.9	60.01 ± 41.21	−12.295 [−37.9; 13.31]	0.012	Small
EXT	Acc	MF (Hz)	174.83 ±16.07	164.01 ± 18.98	−10.827 [−23.63; 1.98]	0.032	Small
EXT	Acc	EMG_mean_ (%)	20.03 ± 10.6	24.39 ± 16.47	4.366 [−6.05; 14.78]	0.003	Small
EXT	Acc	iEMG (AU)	6 ± 3.18	7.31 ± 4.94	1.31 [−1.81; 4.43]	0.005	Small
EXT	Acc	EMG_max_ (%)	49.72 ± 31.63	63.24 ± 34.56	13.528 [−10.36; 37.42]	0.011	Small
FLE	Acc	MF (Hz)	170.54 ± 20.75	162 ± 14.48	−8.542 [−21.26; 4.18]	0.013	Small
FLE	Acc	EMG_mean_ (%)	61.98 ± 23.92	77.47 ± 29.35	15.488 [−4.85; 35.83]	0.028	Small
FLE	Acc	iEMG (AU)	18.6 ± 7.18	23.25 ± 8.81	4.647 [−1.46; 10.75]	0.038	Small
FLE	Acc	EMG_max_ (%)	114.78 ± 35.91	123.54 ± 42.35	8.755 [−20.92; 38.43]	0.004	Small
PEC	Acc	MF (Hz)	121.27 ± 28.65	103.38 ± 21.83	−17.891 [−37.14; 1.35]	0.035	Small
PEC	Acc	EMG_mean_ (%)	72.29 ± 28.38	57.12 ± 27.32	−15.167 [−36.5; 6.16]	0.031	Small
PEC	Acc	iEMG (AU)	21.7 ± 8.52	17.15 ± 8.2	−4.556 [−10.96; 1.85]	0.036	Small
PEC	Acc	EMG_max_ (%)	121.71 ± 40.87	90.33 ± 40.45	−31.387 [−62.58; −0.19]	0.054	Small
TRAP	Acc	MF (Hz)	145.59 ± 36	126.24 ± 23.51	−19.35 [−40.25; 1.55]	0.027	Small
TRAP	Acc	EMG_mean_ (%)	28.28 ± 15.6	28.48 ± 20.55	0.206 [−12.96; 13.37]	0	Small
TRAP	Acc	iEMG (AU)	8.48 ± 4.68	8.54 ± 6.17	0.063 [−3.89; 4.02]	0	Small
TRAP	Acc	EMG_max_ (%)	65.25 ± 29.8	62.12 ± 35.95	−3.133 [−26.78; 20.52]	0.001	Small
TRI	Acc	MF (Hz)	129.62 ± 23.27	122.47 ± 20.89	−7.159 [−23.73; 9.41]	0.006	Small
TRI	Acc	EMG_mean_ (%)	49.04 ± 24.8	54.81 ± 12.72	5.771 [−8.1; 19.64]	0.004	Small
TRI	Acc	iEMG (AU)	14.72 ± 7.45	16.45 ± 3.82	1.733 [−2.43; 5.9]	0.006	Small
TRI	Acc	EMG_max_ (%)	98.9 ± 38.65	100.14 ± 23.64	1.241 [−21.64; 24.13]	0	Small

**Table 3:** Mean ± SD of median frequency (MF), mean EMG (EMG_mean_), integral EMG (iEMG), and maximal EMG (EMG_max_) of Pectoralis Major (PM), Upper Trapezius (UT), Deltoideus Anterior (DA), Deltoideus Medius (DM), Biceps Brachii (BB), Triceps Brachii (TB), Flexor Carpi (FC), and Extensor Carpi (EC) during deceleration phases (Dec).

			Adult	Junior			
Muscle	Phase	Variable	Mean ± SD	Mean ± SD	Difference [95% CI]	Effect Size (η^2^)	Qualitative Inference
BIC	Dec	MF (Hz)	114.02 ± 22.17	108.89 ± 28.01	−5.133 [−23.96; 13.7]	0.004	Small
BIC	Dec	EMG_mean_ (%)	33.8 ± 31.67	45.44 ± 32.14	11.635 [−11.68; 34.95]	0.015	Small
BIC	Dec	iEMG (AU)	6.76 ± 6.34	9.09 ± 6.43	2.329 [−2.34; 7]	0.011	Small
BIC	Dec	EMG_max_ (%)	51.27 ± 41.71	67.09 ± 43.29	15.821 [−15.3; 46.94]	0.014	Small
DANT	Dec	MF (Hz)	141.94 ± 23.63	135.16 ± 18.79	−6.775 [−21.63; 8.09]	0.007	Small
DANT	Dec	EMG_mean_ (%)	43.95 ± 25.78	55.41 ± 20.36	11.458 [−4.7; 27.62]	0.021	Small
DANT	Dec	iEMG (AU)	8.79 ± 5.16	11.08 ± 4.07	2.291 [−0.94; 5.52]	0.014	Small
DANT	Dec	EMG_max_ (%)	66.47 ± 36.31	84.12 ± 31.18	17.657 [−6.08; 41.4]	0.021	Small
DMED	Dec	MF (Hz)	146.25 ± 28.92	130.75 ± 20.68	−15.494 [−33.17; 2.18]	0.029	Small
DMED	Dec	EMG_mean_ (%)	52.25 ± 22.26	45.03 ± 20.57	−7.225 [−22.46; 8.01]	0.009	Small
DMED	Dec	iEMG (AU)	10.45 ± 4.45	9.01 ± 4.11	−1.445 [−4.49; 1.6]	0.005	Small
DMED	Dec	EMG_max_ (%)	78.05 ± 31.64	63.6 ± 30.08	−14.449 [−36.42; 7.53]	0.016	Small
EXT	Dec	MF (Hz)	148.78 ± 16.51	137.52 ± 18.12	−11.263 [−23.77; 1.25]	0.034	Small
EXT	Dec	EMG_mean_ (%)	40.66 ± 31.41	51.48 ± 29.41	10.815 [−10.74; 32.37]	0.017	Small
EXT	Dec	iEMG (AU)	8.14 ± 6.28	10.3 ± 5.88	2.162 [−2.15; 6.47]	0.014	Small
EXT	Dec	EMG_max_ (%)	58.82 ± 41.88	76.56 ± 41.79	17.745 [−12.1; 47.59]	0.018	Small
FLE	Dec	MF (Hz)	166.21± 24.71	156.64 ± 19.37	−9.561 [−25.53; 6.41]	0.016	Small
FLE	Dec	EMG_mean_ (%)	50.45 ± 20.03	67.47 ± 30.2	17.015 [−2.97; 37]	0.034	Small
FLE	Dec	iEMG (AU)	10.09 ± 4.01	13.49 ± 6.04	3.404 [−0.59; 7.4]	0.021	Small
FLE	Dec	EMG_max_ (%)	92.11 ± 25.98	108.75 ± 45.52	16.64 [−12.77; 46.05]	0.016	Small
PEC	Dec	MF (Hz)	125.37± 27.54	111.39 ± 21.11	−13.98 [−32.53; 4.57]	0.022	Small
PEC	Dec	EMG_mean_ (%)	23.97 ± 18.11	24.22 ± 13.86	0.255 [−11.93; 12.44]	0	Small
PEC	Dec	iEMG (AU)	4.79 ± 3.62	4.84 ± 2.77	0.052 [−2.38; 2.49]	0	Small
PEC	Dec	EMG_max_ (%)	57.85 ± 32.54	52.53 ± 23.67	−5.321 [−26.78; 16.14]	0.002	Small
TRAP	Dec	MF (Hz)	160.49 ± 41.71	134.19 ± 28.38	−26.299 [−50.87; −1.73]	0.048	Small
TRAP	Dec	EMG_mean_ (%)	58.64 ± 27.24	52.25 ± 22.88	−6.392 [−23.91; 11.13]	0.007	Small
TRAP	Dec	iEMG (AU)	11.73 ± 5.45	10.45 ± 4.58	−1.281 [−4.79; 2.23]	0.005	Small
TRAP	Dec	EMG_max_ (%)	79.98 ± 34.47	72.33 ± 29.66	−7.647 [−30.08; 14.78]	0.005	Small
TRI	Dec	MF (Hz)	136.9 ± 31.76	136.31 ± 23.48	−0.603 [−20.99; 19.78]	0	Small
TRI	Dec	EMG_mean_ (%)	43.99 ± 32.77	38.5 ± 13.72	−5.489 [−22.95; 11.98]	0.004	Small
TRI	Dec	iEMG (AU)	8.8 ± 6.56	7.7 ± 2.74	−1.099 [−4.59; 2.4]	0.003	Small
TRI	Dec	EMG_max_ (%)	77.06 ± 38.21	73.81 ± 19.91	−3.248 [−24.71; 18.22]	0.001	Small

## Data Availability

Data are contained within the article.
